# Diagnostic and Therapeutic Challenges in Psoriasis–Atopic Dermatitis Overlap: A Retrospective Observational Cohort Study

**DOI:** 10.3390/diagnostics15111381

**Published:** 2025-05-29

**Authors:** Daciana Elena Brănișteanu, Cristina Colac Boțoc, Antonia Elena Huțanu, Cătălina Anca Munteanu, Roxana Paraschiva Ciobanu, Daniel Constantin Brănișteanu, Alin Gabriel Colac, Cătălina Ioana Onu-Brănișteanu, George Brănișteanu, Nicuta Manolache, Elena Porumb-Andrese, Mihaela-Paula Toader

**Affiliations:** 1 Discipline of Dermatology, ‘Grigore T. Popa’ University of Medicine and Pharmacy, 16 Universitatii Str., 700115 Iasi, Romania; daciana.branisteanu@umfiasi.ro (D.E.B.); elena.andrese1@umfiasi.ro (E.P.-A.); 2 Dermatology Clinic, University Clinical Railways Hospital, 1 Garabet Ibraileanu Str., 700115 Iasi, Romania; antoniaclivet@yahoo.com (A.E.H.); anca.munteanu2@yahoo.com (C.A.M.); r.p.ciobanu@gmail.com (R.P.C.); 3 Department of Ophthalmology, ‘Grigore T. Popa’ University of Medicine and Pharmacy, 700115 Iasi, Romania; daniel.branisteanu@umfiasi.ro; 4 Department of Surgery, Oral and Maxillofacial Surgery, Faculty of Dental Medicine, ‘Grigore T. Popa’ University of Medicine and Pharmacy, 700115 Iasi, Romania; colac.alin@yahoo.com; 5 Institute for Cardiovascular Diseases C.C. Iliescu, 258 Fundeni Str., 022328 Bucharest, Romania; catalina.branisteanu@umfiasi.ro; 6 Orthopedy Clinic, Recovery Hospital, 14 Pantelimon Halipa Str., 700661 Iasi, Romania; office_george@yahoo.com; 7 Department of Pharmaceutical Sciences, Faculty of Medicine and Pharmacy, ‘Dunarea de Jos’ University, 800008 Galati, Romania; nicutamanolache@yahoo.com; 8 Discipline of Oral Medicine, Oral Dermatology, ‘Grigore T. Popa’ University of Medicine and Pharmacy, 16 Universitatii Street, 700115 Iasi, Romania; mihaela.toader@umfiasi.ro

**Keywords:** psoriasis, atopic dermatitis, overlap

## Abstract

**Background and Objectives:** Psoriasis and atopic dermatitis (AD) are immune-mediated inflammatory diseases traditionally viewed as distinct. However, a subset of patients may present with overlapping features, leading to diagnostic and therapeutic challenges. This study aims to characterize the clinical, histopathological, and therapeutic features of patients with psoriasis–AD overlap. **Materials and Methods:** A retrospective review was conducted on patients diagnosed with both psoriasis vulgaris and AD between January 2021 and October 2024 at a single tertiary dermatology center. Inclusion required histopathological confirmation of psoriasis and a clinical diagnosis of AD based on Hanifin and Rajka criteria. Clinical features, histopathology, treatment history, and 6-month outcomes were analyzed. **Results:** Out of 469 patients screened, 24 (5.1%) had both conditions. Psoriasis preceded AD in 91.6% of cases. Most patients had intrinsic AD subtypes and moderate-to-severe diseases. Palmoplantar involvement was present in 66.6%, often refractory to biologics alone. Histological overlap complicated diagnosis, with repeated biopsies required in 58.3% of cases. Patients with dual diseases often required combination therapy, and JAK inhibitors showed favorable outcomes in refractory cases. **Conclusions:** Psoriasis–AD overlap represents a distinct clinical entity requiring individualized diagnosis and management. Recognition of this phenotype is critical for optimizing therapeutic outcomes.

## 1. Introduction

Atopic dermatitis (AD) and psoriasis are among the most common chronic inflammatory skin diseases. Both conditions have distinct clinical and immunological features and were historically considered unlikely to coexist. However, emerging evidence suggests a higher than previously believed likelihood of their overlap [1].

AD affects up to 20% of children and 10% of adults, while psoriasis has a global prevalence of 2–3% [2,3]. The true prevalence of psoriasis–AD overlap remains unclear, ranging from 0.2% to 16.5% depending on diagnostic criteria [4,5]. Traditionally considered distinct, increasing data support a bidirectional relationship, with overlapping inflammatory pathways challenging the conventional T helper (Th) 2-driven AD and Th17-driven psoriasis distinction [1,6].

Clinically, psoriasiform lesions may be the only manifestation of AD, while psoriasis can mimic eczema [7]. Diagnosis may require dermoscopy, histopathology, and, in the future, emerging biomarkers like periostin and LL-37 [8]. Overlap cases often involve hand and plantar regions, and many patients present with adult-onset AD rather than a longstanding history of the disease [1,9].

Immunologically, psoriasis is driven by Th1/Th17 cytokines, including Interleukin (IL)-17, IL-23, Interferon-γ (IFN-γ), and Tumor Necrosis Factor-α (TNF-α). In contrast, AD is primarily Th2-mediated (IL-4, IL-5, IL-13). However, chronic AD and some phenotypes involve Th17 and Th22 responses, demonstrating an immune overlap. IL-17 contributes to keratinocyte hyperproliferation in psoriasis and promotes barrier dysfunction in chronic AD. Th17/Th22 pathways also play roles in chronic eczema, suggesting a broader immune interplay [10,11,12].

Psoriasis–AD overlap can manifest in three patterns [13]:

1. Treatment-induced onset—One disease emerges as a side effect of therapy for the other.
2. True overlap syndrome—Both diseases coexist with distinct but simultaneous flares.
3. Sequential occurrence—One disease appears first, followed by the delayed onset of the other.

Although the recognition of psoriasis–AD overlap is increasing, the existing literature remains limited to small studies. This leaves important gaps in our understanding of disease progression and management. Conventional immunosuppressants (methotrexate, azathioprine, cyclosporine) and phototherapy may be effective for both conditions, but treatment must be tailored to individual cases [14].

This study aims to characterize the clinical, histopathological, and therapeutic profile of patients with coexisting psoriasis and AD. We analyzed a cohort of patients with confirmed diagnoses of both conditions. Our goals included examining the disease-onset sequence and the distribution of intrinsic versus extrinsic AD subtypes. We also investigated the role of palmoplantar involvement and the diagnostic limitations posed by overlapping clinical and histological features. Additionally, we examined patterns of treatment response and the need for individualized therapeutic strategies in cases of partial or refractory disease control. Through this detailed analysis, the study provides insight into the complex interplay between Th17- and Th2-mediated inflammation in overlap cases and highlights the unmet diagnostic and therapeutic needs in this unique patient population.

## 2. Materials and Methods

A retrospective, single-center observational study was conducted on patients diagnosed with both psoriasis vulgaris and AD who were evaluated at the Dermatology Clinic of the Clinical Railway Hospital in Iași, Romania, between 1 January, 2021, and 31 October 2024.

During the study period, 316 patients with histopathologically confirmed psoriasis vulgaris and 154 patients diagnosed with AD according to the Hanifin and Rajka criteria were identified. Among these, 24 patients (5.1%) had both conditions concurrently and met the eligibility criteria for detailed analysis.

For each included patient, the following demographic and clinical data were extracted and analyzed: age, sex, disease duration, and baseline and follow-up disease severity scores: PASI (Psoriasis Area and Severity Index) for psoriasis, SCORAD (SCORing Atopic Dermatitis) for AD, and DLQI (Dermatology Life Quality Index) for the life-quality assessment. Where available, additional data on comorbidities (including atopic conditions such as asthma, allergic rhinitis, and conjunctivitis), previous and current treatments, and total serum Immunoglobulin E (IgE) levels were also recorded. No cases of other chronic inflammatory dermatoses or systemic autoimmune diseases were identified in the cohort.

Disease severity was quantified using standardized scoring systems. PASI scores range from 0 to 72 and were classified according to the European Academy of Dermatology and Venereology (EADV) guidelines: mild (PASI ≤ 10), moderate (PASI > 10 and ≤ 20), and severe (PASI > 20). SCORAD scores range from 0 to 103 and were interpreted as follows: mild (<20), moderate (20–40), and severe (>40). DLQI scores range from 0 to 30 and were categorized as: no effect on life (0–1), small effect (2–5), moderate effect (6–10), very large effect (11–20), and extremely large effect (21–30) [15,16,17].

Inclusion Criteria
  - Histopathologically confirmed diagnosis of psoriasis vulgaris.
  - Diagnosis of AD based on Hanifin and Rajka clinical criteria.
  - Availability of documented disease-severity scores: PASI for psoriasis and SCORAD for AD at both baseline and follow-up [15].
Exclusion Criteria
  - Absence of histopathological confirmation for psoriasis.
  - Failure to meet Hanifin and Rajka criteria for AD.
  - Incomplete clinical documentation or missing follow-up data.
Treatment-Response Definitions
  - Complete resolution was defined as a SCORAD score ≤ 10 and a DLQI score ≤ 5 at 6-month follow-up.
  - Significant improvement was defined as a >50% reduction in PASI (for psoriasis) or SCORAD/DLQI (for AD) at 6 months.
  - Partial response was defined as a reduction of 10–15 points in SCORAD and/or DLQI after 6 months of therapy.

## 3. Results

A total of 24 patients with concomitant psoriasis and AD were analyzed. The cohort consisted of 14 females (58.3%, *n* = 14/24) and 10 males (41.7%, *n* = 10/24).

Patients were predominantly older adults, with the largest age group being those over 70 years (29.2%, *n* = 7/24), followed by those aged 60–69 (20.8%, *n* = 5/24). Middle-aged adults (40–49 and 50–59 years) each accounted for 12.5% (*n* = 3/24) of the cohort, while younger adults aged 20–29 made up 12.5% (*n* = 3/24) as well. Adolescents (10–19 years) represented a smaller portion (8.3%, *n* = 2/24), and only one patient was aged 30–39 (4.2%, *n* = 1/24). No patients were under the age of 10 (Table 1).

AD onset varied, skewing toward older age groups. The highest proportion of patients were diagnosed with AD after the age of 70 (20.8%, *n* = 5/24), followed by equal representation in the 20–29 and 30–39 age groups (16.7% each, *n* = 4/24). Patients aged 40–49, 50–59, and 60–69 each accounted for 12.5% (*n* = 3/24) of the cohort. A smaller proportion (8.3%, *n* = 2/24) were diagnosed with AD during adolescence (10–19 years), and no cases were recorded before the age of 10. The age of psoriasis onset among patients with psoriasis–AD overlap was most commonly in mid-adulthood. The largest group experienced disease onset between 40–49 years (25%, *n* = 6/24), followed by those aged 30–39 (20.8%, *n* = 5/24) and 10–19 years (16.7%, *n* = 4/24). Onset during the 20–29 and 50–59 age ranges was observed in 12.5% (*n* = 3/24) of patients each. Fewer patients developed psoriasis after age 70 (8.3%, *n* = 2) or between 60–69 years (4.2%, *n* = 1/24). No cases of childhood-onset psoriasis (under 10 years) were recorded (Table 1).

Analysis of the disease chronology revealed that in the vast majority of cases (91.7%, *n* = 22/24), psoriasis preceded the diagnosis of AD. In the remaining 8.3% (*n* = 2/24) of patients, both conditions were diagnosed simultaneously within the same clinical timeframe. Notably, no patients in the cohort were diagnosed with AD prior to psoriasis (Table 2).

Psoriasis was confirmed histopathologically in all patients included in the study. AD was supported by histological examination in 15 cases (62.5%). Diagnostic uncertainty led to multiple biopsies in a significant proportion of patients. A single biopsy was performed in 41.7% of cases (*n* = 10/24), while 29.2% (*n* = 7/24) required two biopsies and 20.8% (*n* = 5/24) underwent three. In two particularly challenging cases (8.3%), more than four biopsies were necessary to reach a diagnostic consensus (Table 3).

At the time of data collection, the duration of psoriasis varied across the cohort. Over 40% (*n* = 10/24) of patients had lived with psoriasis for more than 10 years, while 37.5% (*n* = 9/24) had disease duration under 5 years, and 20.8% (*n* = 5/24) between 5 and 10 years. In contrast, AD was generally of a more recent onset. The majority of patients (70.8%, *n* = 17/24) had AD for less than 2 years, and only 29.2% (*n* = 7/24) had a disease duration exceeding 2 years.

The intrinsic form of AD was identified in 79.2% of cases (*n* = 19/24), while the extrinsic type was present in 20.7% (*n* = 5/24). Regarding disease severity, mild AD was diagnosed in 16.7% of patients (*n* = 3/24), moderate AD in 58.3% (*n* = 14/24), and severe AD in 29.2% (*n* = 7/24). Atopic stigmata were present in all the patients included in the study (100%, *n* = 24/24). A personal history of atopic comorbidities was reported in 41.7% (*n* = 10/24), while 54.2% (*n* = 14/24) had no such history. To assess the overall burden of atopic comorbidities within the full cohort, we calculated the prevalence each condition relative to the total study population (*n* = 24), rather than only among those affected. Several patients had more than one comorbidity. Allergic conjunctivitis was the most frequent, reported in 33.3% of patients (*n* = 8/24), followed by allergic rhinitis in 29.2% (*n* = 7/24), and asthma in 16.7% (*n* = 4/24). Other allergic conditions, such as food allergies, were noted in 8.3% of patients (*n* = 2/24). Notably, the majority of patients (62.5%) did not exhibit the classical triad of eczema, asthma, and allergic rhinitis. A positive family history of atopic comorbidities and AD was observed in 45.8% of cases (*n* = 11/24) and absent in 54.2% of cases (*n* = 13/24).

Psoriasis severity was classified as mild in 12.5% of cases (*n* = 3/24), moderate in 25% (*n* = 6/24), and severe in 62.5% (*n* = 15/24). Prior to the diagnosis of AD, systemic treatments received by psoriatic patients included methotrexate in 4.2% (*n* = 1/24), an anti-IL-17 agent in 33.3% (*n* = 8/24), and an anti-IL-23 agent in 41.7% (*n* = 10/24). At the time of AD diagnosis, the duration of underlying psoriasis was less than 5 years in 41.7% of patients (*n* = 10/24), between 5 and 10 years in 16.6% (*n* = 4/24), and longer than 10 years in 41.7% (*n* = 10/24). PP involvement was observed in 66.7% of cases (*n* = 16/24).

Histopathological evaluation of the skin biopsies revealed a mixed psoriasiform and eczematous pattern across the cohort. Elongated rete ridges were the most frequently observed feature, present in 100% of cases (*n* = 15/15), followed by agranulosis or hypogranulosis and acanthosis, each identified in 86.7% (*n* = 13/15). Parakeratosis and spongiosis were observed in 80% of cases (*n* = 12/15), along with epidermal exocytosis and perivascular lymphocytic infiltrates, both also seen in 80% (*n* = 12/15). Intraepidermal bullae and superficial dermal vasodilation were noted in 53.3% of cases (*n* = 8/15), while hyperorthokeratosis was present in 46.7% (*n* = 7/15). Langerhans microabscesses were detected in 40% of the cases (*n* = 6/15), whereas classic Munro microabscesses appeared infrequently, found in only 13.3% (*n* = 2/15). Hypergranulosis was identified in 33.3% of cases (*n* = 5/15). Eosinophilic infiltrates in the dermis were not consistently documented in this dataset (Table 4).

All subjects received biologic therapy for psoriasis and achieved complete clearance (PASI = 0) prior to the initiation of AD-specific treatment. In the subgroup that received methotrexate (MTX) for psoriasis, only one patient (4.2%, *n* = 1/24) was concurrently treated with systemic corticosteroids for AD. Of the patients that received biological treatment for psoriasis with an anti-IL-17 agent, 12.5% (*n* = 3/24) received systemic corticosteroids, 8.3% (*n* = 2/24) received a Janus Kinase (JAK) inhibitor and 25% (*n* = 6/24) were not prescribed concomitant systemic therapy for AD. Within the group treated with an anti-IL-23 agent for psoriasis, 20.8% (*n* = 5/24) were prescribed systemic corticosteroids for AD, 25% (*n* = 6/24) received only topical treatment for AD and one patient (4.2%, *n* = 1/24) received a JAK inhibitor (Table 5).

Regarding treatment response in patients with overlapping psoriasis and atopic dermatitis, complete resolution was achieved in 25% of cases (*n* = 6/24) and significant improvement was observed in 41.7% (*n* = 10/24), while a partial response was recorded in 33.3% of patients (*n* = 8/24) (Table 6).

Three representative cases from the cohort highlight key diagnostic and therapeutic challenges encountered in managing psoriasis–AD overlap:

Case 1 involved a 70-year-old female with severe psoriasis and persistent PP lesions, initially treated with guselkumab, followed by secukinumab, due to a lack of improvement. A diagnosis of AD was later confirmed, and disease control was achieved only after the introduction of abrocitinib in combination with secukinumab. This case underscores the role of JAK inhibitors in achieving AD control when biologics alone are insufficient.

Case 2 was a 44-year-old female who developed angioedema and perioral dermatitis following treatment with ixekizumab, prompting its discontinuation. The patient was subsequently managed with bimekizumab for psoriasis and baricitinib for AD, leading to partial clinical improvement. The diagnosis of AD was established based on multiple biopsies and the presence of chronic, lichenified lesions located on the lower legs and feet. Recurrent infections complicated management, highlighting the importance of infection surveillance in patients receiving systemic immunomodulators.

Case 3 described a 63-year-old male with recurrent erythroderma initially treated with systemic corticosteroids and secukinumab. Despite therapy, the patient experienced multiple episodes of erythroderma. A delayed diagnosis of AD was made, and the introduction of baricitinib led to complete remission within 30 days, reinforcing the efficacy of JAK inhibition in severe, treatment-resistant AD presentations.

These cases demonstrate that patients with psoriasis–AD overlap may require individualized therapy, particularly with JAK inhibitors when systemic corticosteroids are insufficient alongside topical treatment. Additionally, infection risk and treatment-associated adverse effects should be carefully monitored in these patients.

## 4. Discussion

**Figure 1:**
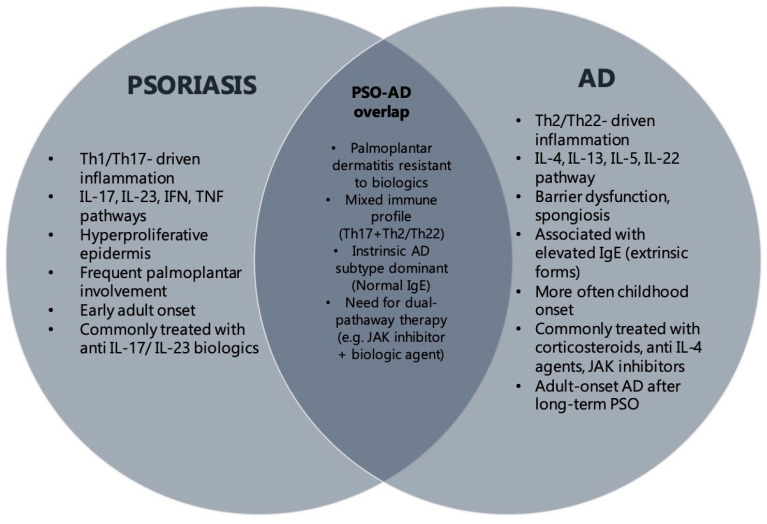
Illustration of the overlapping and distinct clinical and immunological features of psoriasis and AD. The diagram highlights common immune pathways (Th17/Th22) and distinct therapeutic implications.

The coexistence of psoriasis and AD has historically been considered rare due to their opposing immunological pathways. However, emerging evidence challenges this dichotomy. Specific AD subtypes (Asian AD, intrinsic AD, pediatric AD) may exhibit psoriasiform features both clinically and histopathologically [18]. Our findings further support this concept, demonstrating a distinct subgroup of patients with overlapping clinical and histological features. To better illustrate this association, we have provided a visual summary (Figure 1) outlining the shared features of psoriasis and AD. This includes clinical presentation, immune profiles, and therapeutic implications. The schematic aims to clarify the complex interplay between these diseases and provide a concise reference to aid clinical differentiation and management.

In our study, 5.1% of the total cohort (*n* = 469) had concomitant psoriasis and AD, aligning with reports suggesting that the true prevalence may be underestimated due to diagnostic overlap. Age distribution analysis revealed that psoriasis was most frequently observed in the 30–39 age group. AD was more evenly distributed across age groups, suggesting a possible age-dependent disease expression. These findings are consistent with previous studies, such as Egeberg et al., which also found a higher prevalence of adult-onset AD in patients with prior psoriasis history [19]. Additionally, the literature data suggest that psoriatic inflammation may suppress Th2 responses early in life, only for atopic features to later emerge. This may explain why AD was diagnosed significantly later than psoriasis in our cohort [20,21]. Our results support this hypothesis and highlight the importance of long-term monitoring in psoriatic patients for potential atopic manifestations.

PP psoriasis is notoriously refractory to biologic therapies. In our cohort, patients with PP involvement showed lower response rates to IL-17 and IL-23 inhibitors. This aligns with prior reports that PP disease is prone to secondary bacterial colonization and chronic inflammation, which may contribute to therapeutic resistance [22,23]. PP psoriasis was present in 66% of patients, often accompanied by dermatitis affecting the hands and soles. This raises the question of whether PP disease could serve as a clinical marker for psoriasis–AD overlap.

Histopathological findings in our cohort frequently revealed eczematized psoriasis. This diagnosis complicated the differentiation between PP psoriasis and dyshidrotic eczema or hyperkeratotic eczema. The diagnostic ambiguity was further reflected in the high number of biopsies required to establish a definitive diagnosis. In many cases, multiple biopsies were prompted by poor clinical response to psoriasis-targeted therapy, particularly in PP lesions. Despite achieving PASI = 0, persistent or atypical PP lesions raised suspicion for alternative or concomitant pathology. This required additional histological evaluation. Histopathological results were often inconclusive due to overlapping features, showing patterns consistent with eczematized psoriasis, hyperkeratotic eczema, or psoriasiform eczema. Common findings included spongiosis, parakeratosis, variable acanthosis, and mixed dermal infiltrates. However, cases showing marked spongiosis, an increased number of eosinophils, and reduced neutrophilic infiltrates were more likely to be classified as AD-predominant. The subtle patterns suggest that, while helpful, conventional histology alone is insufficient to distinguish true AD from psoriasis variants or paradoxical biologic reaction. This emphasizes the need for more specific diagnostic tools, including immunohistochemical markers or cytokine profiling, such as thymic stromal lymphopoietin or IL-22 staining, to help improve diagnostic accuracy in challenging cases [24].

Histopathological analysis of the overlap cohort revealed a heterogeneous pattern consistent with previously reported findings [23]. Epidermal changes frequently exhibited both psoriasiform and eczematous features, including acanthosis, parakeratosis, elongated rete ridges, spongiosis, intraepidermal vesiculation, and epidermal exocytosis. Dermal alterations were similarly mixed, including perivascular lymphocytic infiltrates and superficial dermal vasodilatation. While classical psoriasis features such as Munro microabscesses were rarely seen, Langerhans-type microabscesses were more commonly observed, suggesting incomplete or altered neutrophilic activity. The coexistence of hypo- and hypergranulosis further emphasized the existence of an overlapping epidermal maturation pattern, rather than a clearly delineated disease entity. These findings align with the recent literature, highlighting that conventional histologic criteria may be insufficient in overlap cases, where lesions may be influenced by concurrent activation of Th1/Th17 and Th2 pathways [7].

An additional challenge relates to the assessment of disease severity using PASI and SCORAD in patients with overlapping diseases. Despite histopathological confirmation, many lesions showed mixed clinical and histological features, making it difficult to attribute them to one disease or the other. This may have resulted in an overestimation of disease burden in one category while underestimating the other. These limitations highlight the need for adjusted or integrated scoring tools tailored to overlap phenotypes. Furthermore, the refractory nature of PP involvement may serve as a clinical clue to underlying overlap. As demonstrated in Case 1, even after multiple biopsies and targeted biologic therapy for psoriasis, along with systemic corticosteroids, JAK inhibitor therapy was required to achieve complete lesion resolution. This suggests that PP lesions in overlap cases may require alternative treatment approaches, particularly dual systemic therapy.

In psoriatic patients, AD can represent either a true comorbid condition or a paradoxical adverse reaction to biological therapy. This distinction is clinically relevant, as it directly impacts therapeutic decisions. True AD typically arises in individuals with an atopic background, and it may be present from the onset of psoriasis or develop later [23]. However, it is important to note that not all diagnostic criteria for AD may be present at the time of initial evaluation. In some cases, clinical signs or supporting biomarkers may emerge gradually, especially in patients already undergoing treatment for psoriasis. This underscores the need for longitudinal observation of psoriatic patients with atopic features, who may later evolve into full-spectrum AD. In our cohort, all patients who developed AD manifestations had atopic stigmata prior to the initiation of systemic therapy for psoriasis. This observation strongly supports the presence of true AD, rather than paradoxical biologically induced eczema. In contrast, paradoxical eczema refers to de novo eczematous eruptions that arise during biological treatment for psoriasis, typically in patients without prior atopic diathesis. Several biologic agents, including TNF-alpha inhibitors, IL-23 and IL-17 inhibitors, have been implicated in such reactions. Distinguishing between true AD and paradoxical eczema can be particularly challenging when clinical and histological findings overlap [25]. In Case 2, the patient presented with both psoriatic plaques and eczematous lesions, leading to diagnostic uncertainty. The eczematous component was initially presumed to be a paradoxical reaction and treated with topical therapies. However, recurrent infections and persistent inflammation limited treatment response. Over time, the persistence of symptoms, along with repeated biopsies and evolving clinical features, supported a diagnosis of concomitant AD. This highlights the clinical burden and quality-of-life impact of misidentification or delayed recognition of dual pathology. In such cases, a comprehensive assessment of atopic history, disease evolution, and histological features is essential to guide appropriate and timely management.

In our study, the intrinsic form of AD was observed in the majority of cases, while only a minority presented the extrinsic subtype. This distribution is noteworthy due to the predominance of Th17/Th22-driven inflammation in intrinsic AD, which partially overlaps with psoriatic immunopathology. This shared immune signature may help explain the coexistence of both conditions in this subset of patients.

Most patients had moderate-to-severe AD, indicating that AD in the context of psoriasis is often clinically significant. Interestingly, despite these findings, only a portion of patients had a personal or family history of atopic comorbidities. Although classical atopic stigmata were observed in all cases, a significant subset lacked the atopic background. This raises the possibility that AD may either emerge independently or be unmasked under certain immunologic pressures, such as biologic therapy or immune system shifts.

Psoriasis severity in this cohort was generally high, with most patients having previously received systemic treatments before the onset of AD. Despite successful control of psoriatic lesions, these patients developed concomitant or subsequent eczematous lesions, further supporting the concept of a distinct overlap phenotype. The timing of diagnoses also suggests a possible sequential pathogenesis. In the majority of cases, AD developed after a prolonged course of psoriasis, supporting the hypothesis that immune modulation may alter disease expression over time. A smaller subgroup also developed AD earlier in the disease course, suggesting that immunologic plasticity could influence the timing and dominance of each component.

An additional observation in our cohort was the apparent inverse relationship between psoriasis and AD severity. Severe psoriasis was more frequently associated with moderate AD, while patients with milder psoriasis were more likely to have more pronounced AD manifestations. This trend may reflect an immunological balancing effect, whereby dominant Th17/Th1-driven inflammation in psoriasis may suppress Th2-mediated pathways and limit the full expression of atopic disease. Similar dynamics have been described in previous studies, supporting the concept that disease severity may shift as immune axes are modulated, particularly during targeted biological therapy [26,27].

Furthermore, the majority of AD cases in this overlap population were classified as the intrinsic subtype, which is characterized by normal IgE levels and Th17/22-driven inflammation. This immunophenotype likely shares greater overlap with psoriasis and may help explain the increased frequency of coexistence in such patients. Although we did not perform cytokine profiling in our cohort, previous studies showed that chronic and intrinsic forms of AD are associated with increased Th17 and Th22 activity. IL-22 and filaggrin dysfunction are shared features, supporting the theory of a mixed immune phenotype. These data suggest that targeting multiple pathways may be particularly effective in managing overlap cases. Collectively, these patterns support the hypothesis that psoriasis–AD overlap represents a unique immunological endotype, rather than a simple coexistence of two unrelated diseases [28].

The relatively low prevalence of asthma and allergic rhinitis in this overlap population may suggest that these patients represent a distinct, possibly non-atopic or mixed immune phenotype of AD. This observation aligns with recent studies proposing heterogeneity within AD, including intrinsic forms that exhibit lower IgE levels, less Th2 dominance, and higher expression of Th1/Th17 pathways [29]. Most patients in our cohort were diagnosed with psoriasis before or at the same time as AD. This pattern does not align with the classical pediatric-onset atopic march. Instead, it indicates that AD in these cases may reflect a distinct, adult-onset inflammatory phenotype. These findings highlight the need for more nuanced phenotyping in patients presenting with features of both diseases. They also point to shared immune mechanisms being involved, rather than sequential or independent disease processes. Interestingly, despite the complex immune background of psoriasis–AD overlap, none of the patients in our cohort had other autoimmune or inflammatory skin diseases such as vitiligo, lichen planus, or multiple sclerosis. This absence may further support the notion that the overlap phenotype observed here is immunologically distinct from broader systemic autoimmunity. It may instead reflect a localized cutaneous immune dysregulation, particularly involving the Th1/Th17/Th22 axis. The lack of systemic autoimmune comorbidities in this subgroup also strengthens the hypothesis that psoriasis–AD overlap represents a unique, skin-limited immune endotype rather than a general autoimmune predisposition.

In all patients included in this study, systemic therapy for psoriasis was initiated prior to any treatment directed at AD. This therapeutic sequencing was based on clinical severity, as psoriasis consistently presented as the more extensive and burdensome condition. Most patients had high PASI scores, widespread lesions, or palmoplantar involvement, necessitating the use of systemic agents such as methotrexate or biologics targeting IL-17 or IL-23 pathways. In contrast, AD symptoms were either subtle, absent, or misclassified initially, becoming clinically significant only after psoriasis was brought under control. This pattern supports the hypothesis that AD may be unmasked or emerge following the suppression of Th17-mediated inflammation, especially in predisposed individuals with intrinsic atopic features.

Among the patients who required JAK inhibitors, all had previously been treated with systemic corticosteroids for AD, without meaningful or sustained improvement. Persistent eczematous lesions, particularly in PP areas or in the setting of recurrent infections, remained refractory despite optimal control of psoriatic disease. The introduction of abrocitinib or baricitinib resulted in significant clinical improvement or complete resolution of AD-related lesions in all three cases. These observations indicate that JAK inhibitors may play a key role in managing the Th2- and Th22-driven inflammatory components of AD, which are not addressed by psoriasis-directed biologic agents. These cases illustrate that, in overlap disease, conventional severity scores (PASI, SCORAD) may fail to fully capture the complexity of dual pathology, and therapeutic decisions often require individualized phenotype-guided approaches. Furthermore, the failure of corticosteroids to achieve adequate control further emphasizes the complexity and treatment resistance of AD manifestations in true overlap cases. Taken together, our findings support a dual-pathway therapeutic strategy, in which JAK inhibitors serve as a critical adjunct to biologics in patients with psoriasis–AD overlap. This association is particularly useful when AD presents with moderate-to-severe features or palmoplantar involvement. Most patients responded adequately to psoriasis-directed biologics, with variable improvement of AD symptoms depending on severity. Mild cases of AD were often controlled with topical therapies alone, whereas more extensive forms required intermittent systemic treatment.

Although JAK inhibitors are primarily approved for AD, recent case reports and emerging data suggest they may also offer therapeutic benefit in psoriasis, particularly in patients with overlapping features. Upadacitinib, a selective JAK1 inhibitor, has demonstrated efficacy in improving psoriatic lesions, including difficult-to-treat areas such as palms and scalp [30,31,32]. These findings indicate a broader role for the JAK-STAT-signaling pathway in psoriatic inflammation. In particular, TYK2, a member of the JAK family, plays a key role in IL-23 and type I interferon signaling, which are central to psoriasis pathogenesis. Deucravacitinib, a selective TYK2 inhibitor, is currently the only JAK-family-targeting agent approved for moderate-to-severe psoriasis, further validating this pathway as a therapeutic target. These observations highlight the possibility that JAK inhibition, particularly in selected phenotypes, may have a broader role than currently recognized, especially in cases of psoriasis–AD overlap [33].

A key limitation of our study is the relatively small sample size (*n* = 24), which restricts the statistical power and generalizability of the findings. Additionally, the observational design may introduce selection bias, limiting the ability to draw causal inferences. Although our cohort represents one of the largest to date with clinically and histologically confirmed psoriasis–AD overlap, it was conducted in a single center, and results may not be representative of broader or more diverse populations.

Another important constraint is the lack of standardized diagnostic criteria for psoriasis–AD overlap, which complicates case classification and contributes to diagnostic variability. While we included clinical, histological, and biomarker data to support diagnoses, immunophenotyping and cytokine profiling were not performed. These techniques could provide more definitive evidence for distinguishing true overlap from paradoxical reactions. Furthermore, treatment regimens varied across patients, reflecting real-world management but limiting controlled comparisons between therapeutic strategies. Despite these limitations, our study provides novel insights into the clinical features, histopathological complexity, and therapeutic challenges of psoriasis–AD overlap. It also highlights the potential role of JAK inhibitors as key agents in refractory or dual-pathway inflammatory disease, warranting further investigation in larger, prospective cohorts.

## 5. Conclusions

The association between AD and psoriasis remains underexplored and continues to raise diagnostic and therapeutic challenges. Although these diseases have traditionally been considered distinct entities, our findings support emerging evidence that a subset of patients exhibit overlapping clinical, histopathologic, and immunologic features. Recent studies suggest that psoriasis–AD overlap may align more closely with psoriasis at a molecular level, with dominant Th17-driven inflammation potentially suppressing Th2 responses until AD-like features emerge. In this context, the development of genomic and immunologic biomarkers may aid in distinguishing true AD from biologically induced eczematous reactions [34,35,36].

Our study emphasizes the need for individualized treatment strategies in patients with overlapping diseases. While biologics remain the cornerstone of psoriasis therapy, patients with coexisting AD, especially those with PP involvement or refractory eczema, may benefit from the addition of JAK inhibitors. Dermatologists should maintain a high index of suspicion for atopic manifestations in long-standing psoriasis, particularly in the presence of treatment-resistant lesions, elevated IgE, or histopathologic findings consistent with eczematized psoriasis or chronic eczema. As research advances, biomarker-driven diagnostics and more precise therapeutic approaches hold promise. However, validation through larger, multicenter studies remains essential to refine disease classification and optimize patient care.

## Figures and Tables

**Table 1:** Demographic characteristics and age at onset of AD and psoriasis in patients with disease overlap.

		*n*/24	%
**Age**	0–9	0	0
	10–19	2	8.3
	20–29	3	12.5
	30–39	1	4.2
	40–49	3	12.5
	50–59	3	12.5
	60–69	5	20.8
	Over 70 years old	7	29.2
**Sex**	Females	14	58.3
	Males	10	41.7
**Age at the time of AD diagnosis**	0–9	0	0
	10–19	2	8.3
	20–29	4	16.7
	30–39	4	16.7
	40–49	3	12.5
	50–59	3	12.5
	60–69	3	12.5
	Over 70 years old	5	20.8
**Age at the time of psoriasis debut**	0–9	0	0
	10–19	4	16.7
	20–29	3	12.5
	30–39	5	20.8
	40–49	6	25
	50–59	3	12.5
	60–69	1	4.2
	Over 70 years olds	2	8.3

AD: atopic dermatitis.

**Table 2:** Timeline of psoriasis and AD in the overlap cohort.

Timeline of the Diagnosis	*n*/24	%
**Psoriasis preceding AD**	22	91.7
**AD preceding psoriasis**	0	0
**Simultaneous onset**	2	8.3

AD: atopic dermatitis.

**Table 3:** Number of skin biopsies required for diagnostic clarification in patients with psoriasis–AD overlap.

Number of Biopsies	*n*/24	%
**1 biopsy**	10	41.7
**2 biopsies**	7	29.2
**3 biopsies**	5	20.8
**More than 4 biopsies**	2	8.3

**Table 4:** Integrated clinical, histopathological, and therapeutic profile of patients with psoriasis and AD overlap.

		*n*/24	%
**Disease duration**			
**Psoriasis**	Under 5 years	9	37.5
	5–10 years	5	20.8
	Over 10 years	10	41.7
**AD**	Under 2 years	17	70.8
	Over 2 years	7	29.2
**Characterization of AD**			
**Type of AD**	Intrinsic	19	79.2
	Extrinsic	5	20.8
**Severity of AD**	Mild	3	12.5
	Moderate	14	58.3
	Severe	7	29.2
**Histopathologically confirmed palmoplantar eczema**	Yes	15	62.5
	No	9	37.5
**Atopic stigmata present**		24	100
**Personal history of atopic comorbidities**	Yes	10	41.7
	No	14	58.3
Atopic comorbidities present *	Allergic conjunctivitis	8	33.3
	Allergic rhinitis	7	29.2
	Asthma	4	16.7
	Other allergies	2	8.3
**Family history of atopic comorbidities**	Yes	11	45.8
	No	13	54.2
**Characterization of psoriasis**			
**Severity of psoriasis**	Mild	3	12.5
	Moderate	6	25
	Severe	15	62.5
**Treatment of psoriasis before the AD diagnosis**	No systemic treatment	5	20.8
	Methotrexate	1	4.2
	Anti-IL-17 agent	8	33.3
	Anti-IL-23 agent	10	41.7
**Disease duration of psoriasis before AD diagnosis**	0–5 years	10	41.7
	5–10 years	4	16.6
	Over 10 years	10	41.7
**Palmoplantar psoriasis**	Yes	16	66.7
	No	8	33.3
**Histopathological features in AD–psoriasis overlap**	**Parameter**	***n*/15**	**%**
	Spongiosis	12	80.0
	Acanthosis	13	86.7
	Parakeratosis	12	80.0
	Intraepidermal bullae	8	53.3
	Hyperorthokeratosis	7	46.7
	Munro microabscesses	2	13.3
	Langerhans microabscesses	6	40.0
	Vascular lymphocytic infiltrate	12	80.0
	Epidermal exocytosis	12	80.0
	Agranulosis/hypogranulosis	13	86.7
	Hypergranulosis	5	33.3
	Elongated rete ridges	15	100
	Superficial dermis vasodilation	8	53.3

AD: atopic dermatitis; IL-17: Interleukin-17; IL-23: Interleukin-23. * Percentages are based on the full cohort. Several patients had more than one atopic comorbidity; thus, totals do not sum 100%.

**Table 5:** Systemic treatments administered for psoriasis and AD in the overlap cohort.

AD Treatment	Systemic Corticosteroids		JAK Inhibitors		No Systemic Therapy for AD	
**Psoriasis treatment**	***n*/24**	**%**	***n*/24**	**%**	***n*/24**	**%**
**Methotrexate**	1	4.2	-	-	-	-
**Anti-IL-17 agent**	3	12.5	2	8.3	6	25
**Anti-IL-23 agent**	5	20.8	1	4.2	6	25

AD: atopic dermatitis; IL-17: interleukin-17; IL-23: Interleukin-23; JAK: Janus Kinase.

**Table 6:** Clinical outcomes at 6-month follow-up in patients with psoriasis–AD overlap.

Outcome in the First 6 Months	*n*/24	%
**Complete resolution**	6	25
**Significant improvement**	10	41.7
**Partial response**	8	33.3

## Data Availability

The original contributions presented in this study are included in the article. Further inquiries can be directed at the corresponding authors.

## Abbreviations

The following abbreviations are used in this manuscript:

AD	Atopic dermatitis
Th1	Type 1 helper T cell
Th2	Type 2 helper T cell
Th17	Type 17 helper T cell
IL-17	Interleukin 17
IL-23	Interleukin 23
IFN-γ	Interferon-gamma
TNF-α	Tumor necrosis factor-alpha
IL-4	Interleukin 4
IL-5	Interleukin 5
IL-13	Interleukin 13
JAK	Janus Kinase
MTX	Methotrexate
SCORAD	SCORing Atopic Dermatitis
PASI	Psoriasis Area and Severity Index
DLQI	Dermatology Life Quality Index
IgE	Immunoglobulin E
EADV	European Academy of Dermatology and Venereology
PP	Palmoplantar
